# Association of Common Genetic Variants in the *CPSF7* and *SDHAF2* Genes with Canine Idiopathic Pulmonary Fibrosis in the West Highland White Terrier

**DOI:** 10.3390/genes11060609

**Published:** 2020-05-30

**Authors:** Ignazio S. Piras, Christiane Bleul, Ashley Siniard, Amanda J. Wolfe, Matthew D. De Both, Alvaro G. Hernandez, Matthew J. Huentelman

**Affiliations:** 1 Neurogenomics Division, Translational Genomics Research Institute, Phoenix, AZ 85004, USA; ipiras@tgen.org (I.S.P.); chrissibleul@gmail.com (C.B.); ashley.siniard@gmail.com (A.S.); a.wolfepack@gmail.com (A.J.W.); mdeboth@tgen.org (M.D.D.B.); 2 Roy J. Carver Biotechnology Center, University of Illinois at Urbana-Champaign, Urbana, IL 61801, USA; aghernan@illinois.edu

**Keywords:** animal genetics, pulmonary fibrosis, genomics

## Abstract

Canine idiopathic pulmonary fibrosis (CIPF) is a chronic fibrotic lung disease that is observed at a higher frequency in the West Highland White Terrier dog breed (WHWT) and may have molecular pathological overlap with human lung fibrotic disease. We conducted a genome-wide association study (GWAS) in the WHWT using whole genome sequencing (WGS) to discover genetic variants associated with CIPF. Saliva-derived DNA samples were sequenced using the Riptide DNA library prep kit. After quality controls, 28 affected, 44 unaffected, and 1,843,695 informative single nucleotide polymorphisms (SNPs) were included in the GWAS. Data were analyzed both at the single SNP and gene levels using the *GEMMA* and *GATES* methods, respectively. We detected significant signals at the gene level in both the cleavage and polyadenylation specific factor 7 (*CPSF7*) and succinate dehydrogenase complex assembly factor 2 (*SDHAF2*) genes (adjusted *p* = 0.016 and 0.024, respectively), two overlapping genes located on chromosome 18. The top SNP for both genes was rs22669389; however, it did not reach genome-wide significance in the GWAS (adjusted *p* = 0.078). Our studies provide, for the first time, candidate loci for CIPF in the WHWT. *CPSF7* was recently associated with lung adenocarcinoma, further highlighting the potential relevance of our results because IPF and lung cancer share several pathological mechanisms.

## 1. Introduction

Canine idiopathic pulmonary fibrosis (CIPF) is a chronic and progressive fibrotic lung disease that particularly affects the West Highland White Terrier dog breed (WHWT) [1]. CIPF shares several clinical and pathological features with human IPF and it has been proposed as a possible sporadic disease model [2]. A typical clinical feature occurring in the majority of affected dogs is inspiratory crackles, as well as laryngo-tracheal reflex, tachypnea, and excessive abdominal breathing [2,3]. Human IPF is considered to be a disease of the epithelium. Specifically, microscopic injuries of the aging lung epithelium lead to defective regeneration and abnormal epithelial–mesenchymal crosstalk with activation of transforming growth factor beta (TGF-β) [4,5]. This is followed by extravascular coagulation, immune system activation, and secretion of excessive amounts of extracellular matrix (ECM) proteins [2]. However, the overall initiating cause of this pathological cascade in dogs and humans is still unknown.

Several studies have attempted to clarify the molecular mechanisms of CIPF in WHWTs. Maula et al. [6] reported the upregulation of activin A in the lung alveolar epitelium of WHWTs with CIPF. Furthermore, increased TGF-β1 signaling activity was detected in WHWTs and other predisposed breeds (such as Scottish Terriers and Bichons Frise) compared to non-predisposed breeds [7]. In an RNA expression profiling study in dogs, chemokine and interleukin genes were found to be overexpressed in the lungs, with *CCL2* mRNA levels also noted as being elevated in serum [8]. Endothelin-1, measured in both serum and bronchoalveolar lavage fluid, has been suggested to be a biomarker suitable to differentiate dogs with CIPF from dogs with chronic bronchitis and eosinophilic bronchopneumopathy [9]. 

Genetic background is considered one of the risk factors for both CIPF and IPF [3,10]. Genome-wide association studies (GWAS) have led to the detection of several genes linked with the disease in humans. Specifically, three GWAS have been conducted in humans detecting signals in *AKAP13*; *MUC5B*; *DSP*; *TOLLIP*; *MDGA2*; *SPPL2C*; and *TERT* [10,11,12]. However, genetic risk factors for CIPF in the WHWT have not been identified. The domestic dog (*Canis familiaris*) is a useful model for many human diseases [13] due to the high number of analogous diseases [14], similar physiologies and medical care, as well as the simplified genetic architecture in purebred dogs [15]. Each dog breed originated from a small founder population with consequently low levels of genomic heterogeneity and long stretches of linkage disequilibrium (LD). Due to these characteristics, GWAS in dogs have increased statistical power comparable to, or better than, those performed in human population isolates [16]. This genetic homogeneity, the consequence of strong artificial selection conducted by humans, also led to an excess of inherited diseases, offering unique opportunities to discover genetic associations for spontaneous diseases [13]. Several GWAS have been conducted in specific breeds using the canine single nucleotide polymorphisms (SNP) array leading to the discovery of genetic risk factors for ectopic ureters [17], inflammatory bowel disease [18], hereditary ataxia [19], and hypothyroidism [20], among others.

In this study, we conducted, for the first time, a GWAS in a sample WHWTs with CIPF and unaffected controls using whole genome sequencing and imputation with the goal of finding genetic risk factors that may predispose the WHWT to the disease.

## 2. Methods

### 2.1. Sample Collection

A total of 73 dogs, including 28 affected (AF) and 45 unaffected (UF), were sampled via internet-based recruitment of saliva samples (the study site can be found at www.tgen.org/westie). Participants were directed to self-report the breed and diagnostic status of their dog. Other clinical information was not available for this study. The complete enrollment form can be found in File S1. The study protocol was reviewed and approved by TGen’s Institutional Animal Care and Use Committee (protocols #13055, #15002, #18006) in accordance with relevant guidelines and regulations. Owner consent for collection of the samples used in this study was obtained. The sex ratio (male/females) in AF dogs was 0.86, whereas in UF dogs it was 1.26 (*p* = 0.466). The average age for AF dogs was 12.8 years (range: 6.9–17.0), whereas in UF dogs it was 12.7 years (range: 9.1–16.8) (*p* = 0.793). Sex information was not available for four samples, and age information was not available for one sample. Saliva was collected by the owner using the Oragene ANIMAL kit (DNAGenotek, Ottawa, CA) and returned to the laboratory at ambient temperature. 

### 2.2. DNA Extraction and Whole Genome Sequencing

DNA was isolated from the collected saliva specimens according to the manufacturer′s instructions. Construction of the shotgun genomic libraries and sequencing on the NovaSeq 6000 was carried out at the Roy J. Carver Biotechnology Center, University of Illinois at Urbana-Champaign. DNA was quantitated with the Qubit High Sensitivity reagent (Thermo Fisher, Waltham, MA) and diluted with water to 2.5 ng/μL in a total volume of 12uL. Libraries were prepared with the Riptide DNA library prep kit (iGenomX, Carlsbad, CA). Briefly, random primers with 5′ barcoded Illumina adapter sequences (one sequence that is unique to each sample) were annealed to denatured DNA template. A polymerase extended each primer and this action was terminated with a biotinylated dideoxynucleotide, of which there was a small fraction in the nucleotide mix. The biotinylated products were then pooled for all of the samples and captured on streptavidin-coated magnetic beads. A second 5′ adapter-tailed random primer was used with a strand-displacing polymerase to convert the captured DNA strands into a dual adapter library. PCR was used to amplify the products and add an index barcode. These libraries were then sequenced for 150 nt from each side of the DNA fragments (paired-reads) on a NovaSeq 6000 (Illumina, San Diego, CA, USA) one lane of an S2 flowcell. 

### 2.3. Data Analysis

The fastq files were generated with the *bcl2fastq v2.20* Conversion Software (Illumina) and demultiplexed with the fgbio tool from Fulcrum Genomics (https://github.com/fulcrumgenomics/fgbio). Sequencing reads were processed and imputed using version 2.0 of the Gencove, Inc. analysis pipeline for canine low-pass sequencing data. Reads were aligned to the reference genome *CanFam3.1* using *bwa mem v0.7.17* [21] and sorted, and duplicates were marked using *samtools v1.8* [22], and imputation performed using *loimpute v0.18* (Gencove, Inc.), on the basis of the model of Li et al. [23]. The imputation reference panel consisted of 676 sequenced dogs across the 91 dog breeds for a total of 53 million sites.

The resulting *vcf* files of 28 AF and 45 UF dogs were filtered using *vcftools v0.1.16* [24] including only biallelic, single nucleotide variants (SNVs) and variants with genotype probability (GP, indicating the imputation quality) greater or equal then 0.90. Then, we filtered the dataset with *PLINK v1.9* [25] using the following thresholds: SNP genotyping rate ≥95%, minimum allele frequency (MAF) ≥5%, Hardy–Weinberg equilibrium in unaffected *p* ≥ 1.0 × 10^−5^, sample genotyping rate ≥90%, and keeping only autosomal variants. We conducted principal component analysis (PCA) with *PLINK v1.9* to detect and remove outliers. Specifically, we used the identity by similarity (IBS) metric taking into account from the first to the fifth closest neighbor, and classifying as outliers samples with *Z* ≤ −4, representing 4 standard deviations below the group mean. After outlier removal, the original dataset including only high-quality imputed SNPs was filtered again with *PLINK v1.9* using the same thresholds. Identity by descent (IBD) analysis was conducted to estimate the relatedness between all the pair of samples calculating the *pi-hat* value using the *--genome* command in *PLINK v1.9*. This analysis was conducted as additional quality control (i.e., identification of duplicated samples), and the adjustment for relatedness in the GWAS was conducted using the relatedness matrix computed with *GEMMA v0.96* [26,27] (see below).

The GWAS was conducted using a mixed linear model (MLM) to account for relatedness and population structure, as implemented in the *GEMMA v0.96* software, assessing the significance with the Wald test. The first step of the analysis included the estimation of the relatedness matrix, and the top 10 principal components. These metrics were included in the second step (GWAS), allowing for the adjustment for both relatedness and population structure. Results were corrected at the genome-wide level using the Bonferroni method, accounting for the number of independent SNPs tested according the linkage disequilibrium (LD) patterns estimated using the option *--indep-pairwise* 10,000 1 0.80 in *PLINK v1.9*. Using this approach, we found 101,740 independent SNPs. Variants were annotated according *CanFam3.1* assembly using the R-package *Biomart v2.42.0* [28]. Lambda inflation factor (*λ*) and quantile-quantile plots (qqplots) were computed using the R-package *snpStats*. 

We further analyzed the data, conducting a gene-based association analysis using the *GATES* method [29], using as input the summary statistics obtained from the *GEMMA* analysis. First, we filtered the dataset including the SNPs located at ± 1500 bp from each gene to include the variants located in the promoter and in the 3′ regions. The analysis was also conducted including larger regions (± 5000 and ± 10,000 bp). Ensemble start and end coordinates of each gene were retrieved using the R-package *Biomart v2.42.0*, according the dog assembly *CanFam3.1*. The Ensembl Biomart gene coordinates correspond to the outermost transcript start and end. Then, for each gene, we computed a matrix of correlation between SNPs using the unaffected samples in order to account for the LD. The correlation matrix was computed with the Pearson’s method using the *cor* function implemented in R, using the option *use = “na.or.complete”* to deal with missing genotypes. Finally, the *GATES* statistics were computed using as input the *p*-values from the MLM analysis and the correlation LD matrix. The analysis was conducted using the *GATES2* function as implemented in the R-package *aSPU* (https://cran.r-project.org/web/packages/aSPU/index.html). *p-*values were corrected using the Bonferroni method adjusting for the total number of genes tested.

Results from the both SNP and gene level analysis were compared with a list of 41 genes compiled from the largest and most recent human IPF GWAS [30,31]. Allele frequencies were compared using a general dog population reference dataset including several breeds [32].

## 3. Results

### 3.1. Filtering and Quality Controls 

After imputation, we removed INDELS, low quality variants, and variants with more than two alleles. We obtained a median number of SNVs per sample equal to 35,916,311 (range: 33,918,432–36,243,510). Samples showed a median depth of 1X. The median value of variants covered with equal to or greater than five reads per sample was 1,902,261 (range: 34,490–12,200,995) (Table S1). We filtered the dataset with *PLINK v1.9*, obtaining 1,839,683 variants for study with an average genotyping rate equal to 98.0%. We conducted PCA and IBS analyses to identify significant outliers and identified one outlier, on the basis of the first and second principal components, with a Z score < −4 (Figure S1). We removed this sample from the analysis and we filtered the dataset again, obtaining 1,843,695 SNPs in 28 AF and 44 UF animals. We re-ran the PCA and IBS analyses and did not identify any additional outliers. AF and UF groups were not statistically different for sex (*p* = 0.466) or age (*p* = 0.793). The IBD analysis demonstrated a *pi-hat* = 0.043 ± 0.068 (range: 0.000–0.500). The distribution of *pi-hat* values for each sample pair is reported in Figure S2.

### 3.2. Genome-Wide Association Study

**Figure 1:**
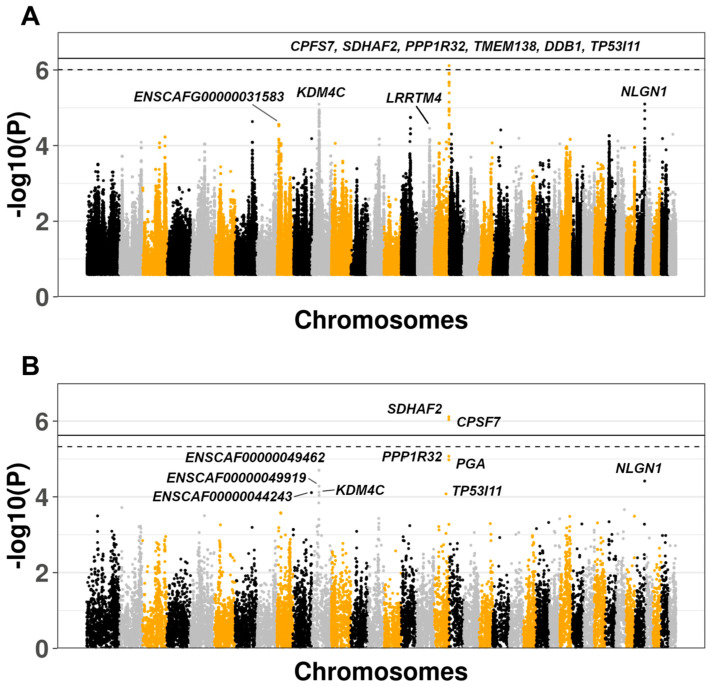
(**A**) SNP level analysis: Manhattan plot of the top 500K SNPs ranked by unadjusted *p*-value. The continuous and dashed lines indicate the genome-wide (*p* < 4.91 × 10^−7^) and suggestive (*p* < 9.83 × 10^−7^) significance thresholds, respectively. The *p*-value adjustment was conducted using the Bonferroni method, accounting for 101,740 independent SNPs estimated using regional linkage disequilibrium (LD) patterns. Gene names reported are the top 10 according to the SNP level analysis. (**B**) Gene level analysis: Manhattan plot of all the genes ranked by *p*-value. The continuous and dashed lines indicate the genome-wide and suggestive significance thresholds, respectively. The adjustment was conducted using the Bonferroni method accounting for the total number of genes tested (*n* = 18,110). Gene names reported are the top 10 according the gene level analysis.

We ran the GWAS accounting for relatedness and population stratification using the linear mixed model as implemented in the *GEMMA* software [26,27]. Age and sex were not included as covariates in the model because these factors did not differ significantly between the AF and UF animals. The results were adjusted (adj) with the Bonferroni method accounting for the 101,740 independent SNPs estimated by regional LD patterns, setting the genome-wide significance threshold at *p* < 4.91 × 10^−7^ (adj *p* < 0.05). We considered a level of *p* < 9.83 × 10^−7^ (adj *p* < 0.10) as “suggestive” association. The top 10 variants ranked by adj *p*-value are reported in Table 1, and the Manhattan plot with the top 500K SNPs is illustrated in Figure 1A.

**Figure 2:**
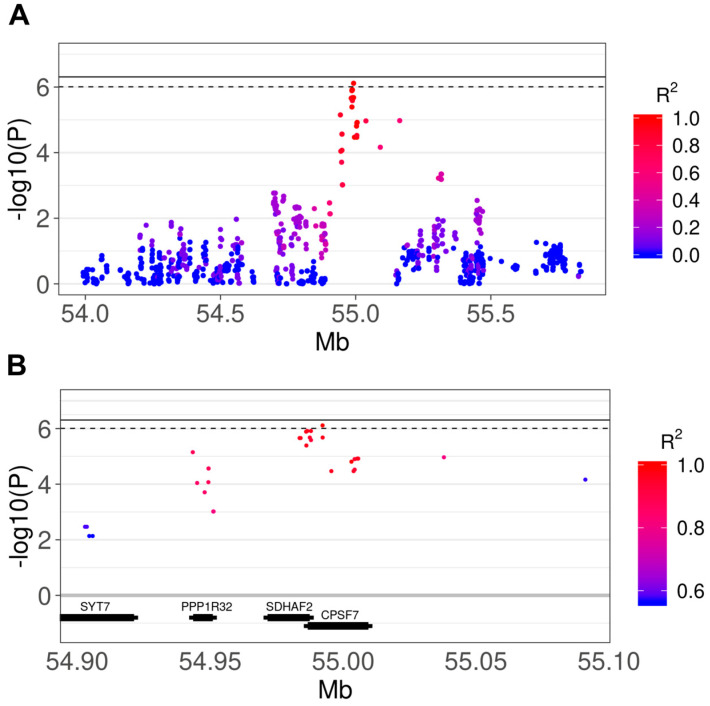
Manhattan plots showing the details of the association region in chromosome 18. The continuous and dashed lines indicate the genome-wide and suggestive significance thresholds, respectively. The color of the points indicates the LD (expressed as *R^2^*) between the top (rs22669389) and the close SNPs. Values of *R^2^* range from 0 (absence of LD) to 1 (complete LD). Figure 2B shows a smaller *R^2^* range due to the closeness of the top SNP. (**A**) Region ± 1 Mb from the top SNP; (**B**) region ± 0.1 Mb from the top SNP. Thick sections of the genes represent the actual gene region according to Ensemble, the thin sections represent the surrounding regions (± 1500).

We obtained *λ* = 1.052, demonstrating an absence of significant population stratification after principal components adjustment (Figure S3). We did not detect any genome-wide significant variant after multiple testing correction, but one variant (rs22669389) was identified at a suggestive level of significance (adj *p* = 0.078). All of the top SNPs reported in Table 1 are located in the same region on chromosome 18 between 54,983,627 and 54,992,285 (8658 bp), encompassing the two overlapping genes succinate dehydrogenase complex assembly factor 2 (*SDHAF2*) and cleavage and polyadenylation specific factor 7 (*CPSF7*), and being located upstream, in introns or in 5’ untranslated regions (UTR) of the two genes. In addition to the SNP-level-analysis, we computed a multi-marker test using the *GATES* method, adjusting the results for the total number of genes tested (*n* = 18,110; *p* < 2.76 × 10^−6^). SNPs were assigned to a gene when ± 1500 bp from the gene was found. The results showed two significant genes after Bonferroni correction: *CPSF7* (adj *p* = 0.016) and *SDHAF2* (adj *p* = 0.024). The corresponding Manhattan plot is shown in Figure 1B. The results of the *GATES* analysis were confirmed when we considered larger distances for the SNP to gene assignments (for both ± 5000 bp and ± 10,000 bp). The regional plot including all of the SNPs in the region is shown in Figure 2, also reporting the LD patterns as *R^2^* values. Figure 2A shows a region of 2 Mb around the top SNP (rs22669389), with the *R^2^* ranging from 0 (absence of LD) to 1 (complete LD). Figure 2B shows a smaller region around the top SNP (0.2 Mb), showing the *R^2^* ranging from 0.56 to 1. 

### 3.3. Comparison of Results With Human IPF GWAS and Canine SNP Reference Data

We considered the two largest and most recent human IPF GWAS to compile a list of 41 candidate genes and compare our results [30,31]. The list included signals detected in the two studies, as well as genes identified in previous studies tested for validation purposes. From our results, we included all SNPs or genes (*GATES* analysis) with *p* < 0.05, (*n* = 104,370 SNPs), and a number of genes ranging from 2614 to 3616, depending on the gene flanking region used (± 1500 bp, ± 5000 bp, and ± 10,000 bp). We detected nine genes showing SNPs with *p* < 0.05, and nine genes showing at least one significant result in the GATES analysis, five of them overlapping with the SNP analysis (*CD1C*, *DEPTOR*, *MAD1L1*, *MRPL13*, and *MUC5B*). All the genes in our study showed *p* < 0.05, with the exception of *MUC5B* (*p* < 0.01) (Table S2).

Allele frequencies of the top SNPs in Table 1 were compared with a reference dataset generated for imputation purposes, including a whole genome data from 365 dogs from different breeds [32]. We observed the allele frequencies of non-WHWT (*n* = 362) as similar to the affected dogs in our study, with the exception of one SNP. Additionally, allele frequencies of the WHWT (*n* = 3) were in the range of average allele frequency in our study (Table S3). However, the sample size of this reference dataset is likely too small to estimate an accurate allele frequency within each breed.

## 4. Discussion and Conclusions

We were able to detect significant genetic risk factors for CIPF in the West Highland White Terrier dog breed using a GWAS including 1,839,683 informative SNPs. Applying a gene-level approach, we observed genome-wide significant signals in *CPSF7* (cleavage and polyadenylation specific factor 7) and *SDHAF2* (succinate dehydrogenase complex assembly factor 2) (adj *p* = 0.016 and adj *p* = 0.024, respectively). These two overlapping genes include 15 and 8 SNPs, respectively. All of the top associated SNPs were located in introns, 5’UTR, or upstream of the two genes. 

*CPSF7* is a human orthologue (gene order conservation score = 100), and encodes for the 59 kDa subunit of *Cleavage Factor Im*, involved in the cleavage and polyadenylation of pre-mRNAs. It is related to several mRNA process pathways, such as “mRNA splicing”, “metabolism of RNA”, “mRNA 3′-end processing”, “processing of capped intronless pre-mRNA”, and “RNA polymerase II transcription”. In a recent study, *CPSF7* was found to be involved in lung adenocarcinoma (LAD). Specifically, Sp1 Transcription Factor (SP1) induces the promoter activity of *LINC00958,* which, when overexpressed, drives LAD progression via the miR-625-5p/CPSF7 axis [33]. The genetic association reported in our study might reveal the importance of CPSF7 in CIPF, perhaps through the same pathologic mechanism as in lung cancer. IPF in humans is a risk factor for lung cancer, increasing the chance of development from 7% to 10% [34]. Additionally, there are several genetic, molecular, and cellular mechanisms shared between lung fibrosis and lung cancer such as myofibroblast activation, endoplasmatic reticulum stress, alteration of growth factor expression, and genetic and epigenetic variations [34]. *CPFS7* has also associated with liver cancer [35].

*SDHAF2* encodes a mitochondrial protein involved in the flavination of a succinate dehydrogenase complex subunit and it has largely been associated with paragangliomas in previous literature [36,37].

We compared our results with the two largest and most recent human IPF GWAS [30,31], finding in our study a total of 13 genes with *p* < 0.05 that overlapped with the human candidate gene list. Five genes were detected at both the SNP and gene level analyses (*CD1C*, *DEPTOR*, *MAD1L1*, *MRPL13,* and *MUC5B*). However, all of the genes showed a weak significance (*p* < 0.05), other than mucin 5B, oligomeric mucus/gel-forming (*MUC5B*; *p* < 0.01).

This study presents some limitations. First, the disease was self-reported by the owners, and thus we did not have any clinical confirmation of CIPF, knowledge of whether they had progressive lung fibrosis, or information about lifespan of the cohort. Second, the average age of diagnosis for CIPF in WHWTs is between 9 and 13 years of age [1,38,39]. Our unaffected samples had an average age of 12.7 years (range: 9.1–16.8), with 12 samples (27.3%) that were 14 years of age and older. Therefore, it is possible that some of our declared “unaffected” dogs may develop CIPF in the future.

In conclusion, we report for the first time the identification of genetic variants associated with CIPF in the West Highland White Terrier dog breed, located in a region encompassing the *CPFS7* and *SDHAF2* genes. Our findings demonstrated some overlap with biological functions, with compelling links to previously demonstrated findings in lung cancer, sharing several biological and genetic features with IPF in humans.

## Figures and Tables

**Table 1:** Details of the top 10 single nucleotide polymorphisms (SNPs) detected in the genome-wide association study (GWAS). *p*-values were adjusted using the Bonferroni method, accounting for 101,740 independent SNPs.

Refsnp ID	CHR	BP	A1	A2	F_A	F_U	Depth (SD)	Beta	*p*	Adj *p*	Ensembl Gene ID	Gene Name	Consequence Type
rs22669389	18	54992254	T	A	0.704	0.333	0.451 ± 0.713	0.406	7.7 × 10^−7^	0.078	*ENSCAFG00000030303; ENSCAFG00000016152*	*SDHAF2; CPSF7*	u, i; u, i
rs22647286	18	54987884	C	T	0.704	0.333	3.183 ± 2.875	0.394	1.2 × 10^−6^	0.124	*ENSCAFG00000030303; ENSCAFG00000016152*	*SDHAF2; CPSF7*	u, i; u, i
rs851654341	18	54986491	A	G	0.704	0.333	2.324 ± 2.123	0.394	1.2 × 10^−6^	0.124	*ENSCAFG00000030303; ENSCAFG00000016152*	*SDHAF2; CPSF7*	u, i; u, i
rs852097932	18	54986070	A	G	0.704	0.337	2.861 ± 2.209	0.394	1.3 × 10^−6^	0.131	*ENSCAFG00000030303; ENSCAFG00000016152*	*SDHAF2; CPSF7*	u, i; u, i
rs22686152	18	54992285	A	G	0.704	0.345	0.732 ± 0.940	0.386	2.1 × 10^−6^	0.213	*ENSCAFG00000030303; ENSCAFG00000016152*	*SDHAF2; CPSF7*	u, i; u, i
rs22647289	18	54987464	G	T	0.704	0.345	5.423 ± 3.702	0.391	2.1 × 10^−6^	0.214	*ENSCAFG00000030303; ENSCAFG00000016152*	*SDHAF2; CPSF7*	5’ UTR, u; 5’ UTR, u
rs850942449	18	54983627	A	G	0.704	0.345	2.831 ± 2.449	0.393	2.2 × 10^−6^	0.223	*ENSCAFG00000030303; ENSCAFG00000016152*	*SDHAF2; CPSF7*	u, i; u, i
-	18	54984004	G	A	0.704	0.345	0.887 ± 0.919	0.393	2.2 × 10^−6^	0.223	*ENSCAFG00000030303*	*SDHAF2*	i
rs22647283	18	54987912	C	T	0.692	0.326	2.535 ± 2.709	0.390	2.6 × 10^−6^	0.263	*ENSCAFG00000030303; ENSCAFG00000016152*	*SDHAF2; CPSF7*	u, i; u, i
rs850871193	18	54986170	C	T	0.692	0.337	3.028 ± 2.646	0.387	4.1 × 10^−6^	0.413	*ENSCAFG00000030303; ENSCAFG00000016152*	*SDHAF2; CPSF7*	u, i; u, i

A1: minor frequency allele referred to the total sample; A2: major frequency allele referred to the total sample; AF: frequency of A1 in affected; UF: frequency of A2 in unaffected; u: upstream; i: intron; 5’ UTR: 5’ untranslated region; *SDHAF2*: succinate dehydrogenase complex assembly factor 2; CPSF7: Cleavage And Polyadenylation Specific Factor 7.

## Supplementary Materials

The following are available on line at www.mdpi.com/xxx/, File S1: Informed consent for biological sample collection. Table S1. Median number of SNPs at different depth levels before quality control filtering. Table S2. Comparison of the list of genes compiled from two large human GWAS investigating IPF; Table S3. Comparison of the allele frequencies of the 10 top SNPs in the GWAS with data from Hayward et al. [32] including 365 samples from different breeds. Figure. S1. Scatterplot of principal components 1 and 2 of all of the sequenced samples before outlier removal. Figure. S2. Distribution of the *pi-hat* value computed between each pair of dogs included in the GWAS. Figure. S3. QQplot showing the observed and expected distribution of GWAS *p*-values.
