# Supplementary material for: Association of Common Genetic Variants in the CPSF7 and SDHAF2 Genes with Canine Idiopathic Pulmonary Fibrosis in the West Highland White Terrier
Source: Genes (Basel). 2020 May 30;11(6):609. doi: 10.3390/genes11060609 (PMC7349241; doi:10.3390/genes11060609)
Supplement: Supplementary file 1 [file genes-11-00609-s001.zip › Supplementary/Figure S1-S3.docx]

**
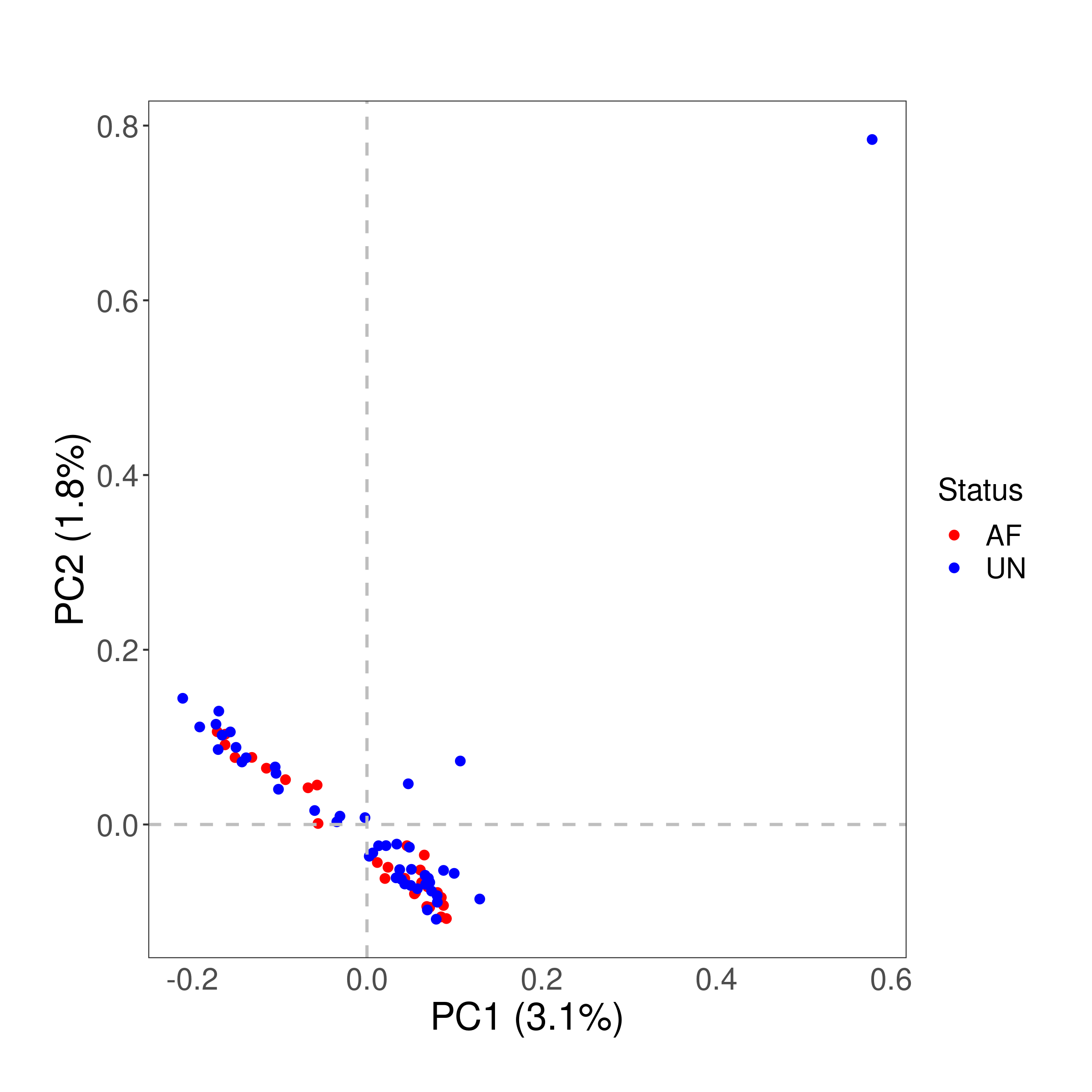
**

**Figure S1**. Scatterplot of the Principal Component 1 and 2 of all the sequenced samples before outlier removal.


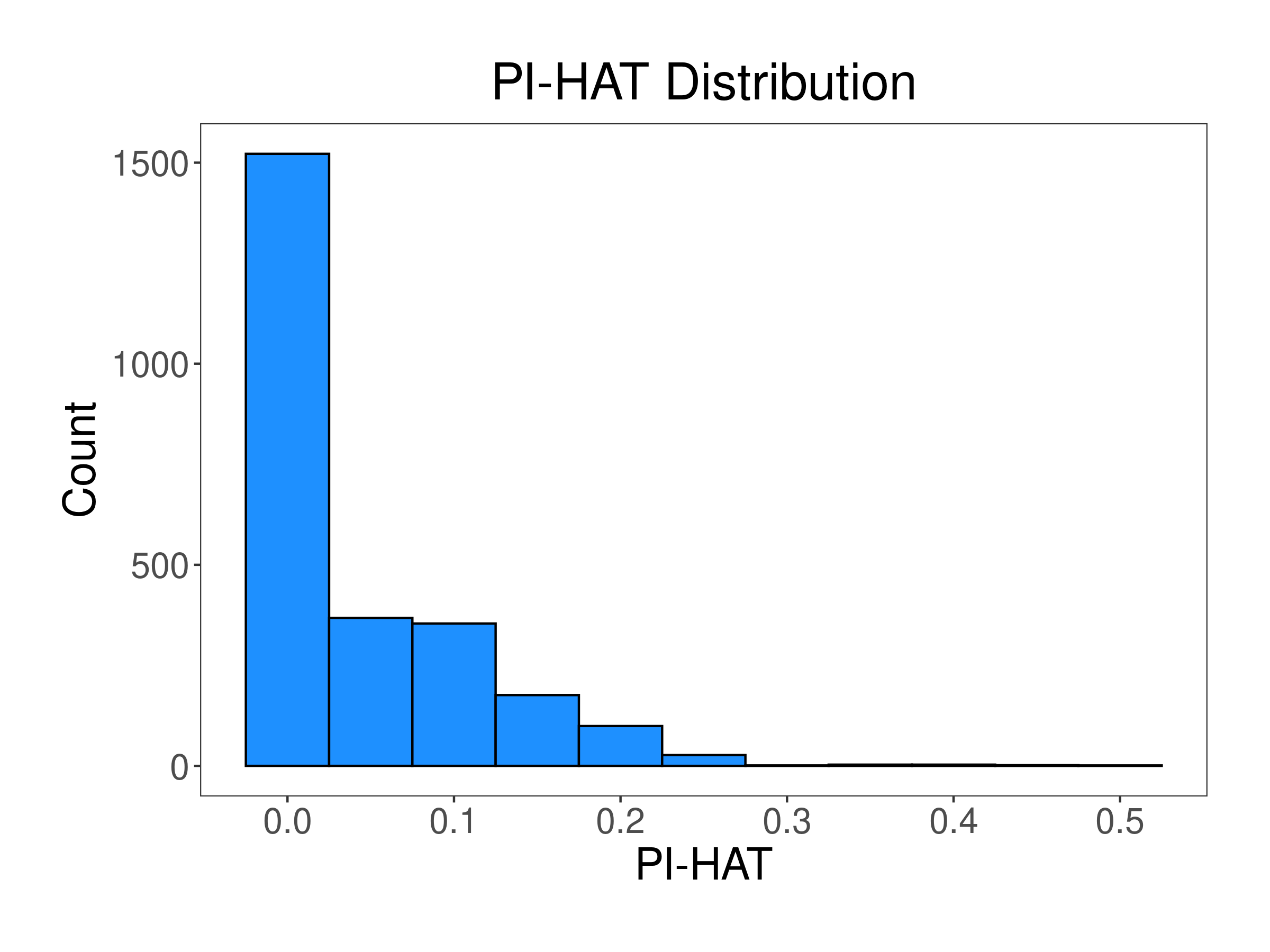


**Figure S2.** Distribution of the pi hat value computed between each pair of dogs included in the GWAS.

**
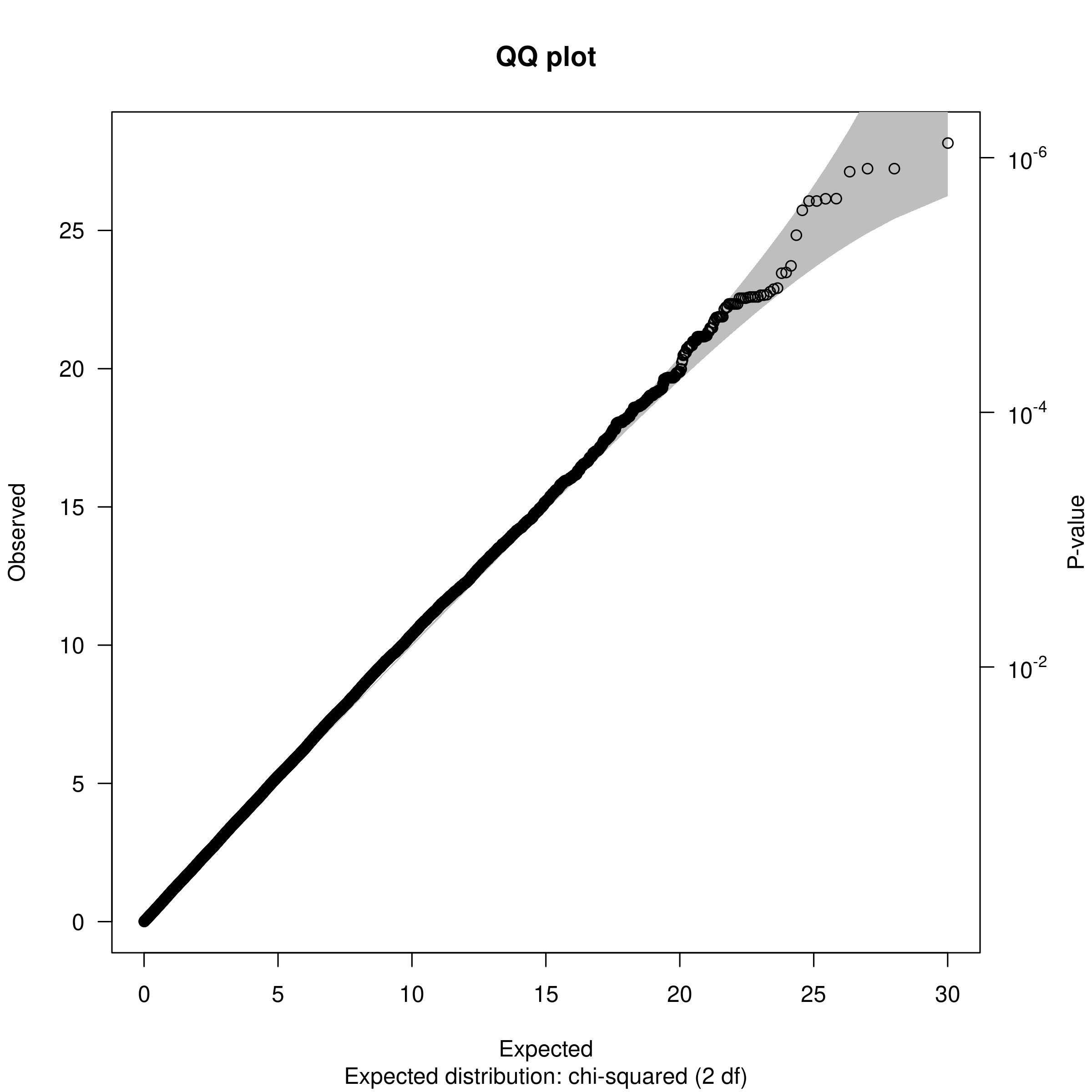
**

**Figure S3**. QQplot showing the observed and expected distribution of GWAS p-values.
