# Supplementary material for: Association of Common Genetic Variants in the CPSF7 and SDHAF2 Genes with Canine Idiopathic Pulmonary Fibrosis in the West Highland White Terrier
Source: Genes (Basel). 2020 May 30;11(6):609. doi: 10.3390/genes11060609 (PMC7349241; doi:10.3390/genes11060609)
Supplement: Supplementary file 1 [file genes-11-00609-s001.zip › Supplementary/File_S1.pdf]

v 1.0

OWNER CONSENT
CANINE GENETICS & GENOMICS

Canine Saliva Collection as Essential Material for Genetic Analysis

Matthew J. Huentelman, PhD

I voluntarily elect to enroll my pet in a program where a sample of saliva and/or an oral cavity swab is obtained principally for DNA isolation to advance canine research. The DNA will be utilized to better understand lung disease that is prevalent in the West Highland White Terrier. Your dog's DNA sample may be collected either because your dog has been diagnosed with lung disease, because your dog is older than a certain age and has never been diagnosed with lung disease, or because your dog is from a breed that is closely related to the West Highland White.

As a voluntary participant, I have been appraised, do understand, and agree to the following.

1. I am fully aware of the risks, side effects, benefits, and consequences of collection.

2. I authorize photography for documentation (if needed), and agree to allow broad use of information gathered from my pet in scientific communications, publications, reports and/or presentations to institutional, governmental, and sponsoring agencies charged with overseeing this research program. I further understand **all personal information will be kept strictly confidential.**

3. I have been given the opportunity to ask questions regarding sample collection; all such questions have been answered to my satisfaction.

After reading the above, I voluntarily consent to my dog(s) participating in this study.

____

Owner (Print)

____

Owner (Signature) **Date**

The Translational Genomics Research Institute
445 N. 5th Street
Phoenix, AZ 85004
(602) 343-8400

Owner/Agent Information

Name:____

Address:____

City:_______________________State:______Zip:_______________

Phone:____

Email:____

Canine Information

Call Name and/or Registered Name:____________________________

Reg. # (if available):____

Date of Birth:____

Sex (Circle one): MI MN FI FS

Breed:____

Coat Color:____

Please provide any additional info you believe may be helpful (health history, etc):

The Translational Genomics Research Institute
445 N. 5th Street
Phoenix, AZ 85004
(602) 343-8400
